# Reduced Glutathione: A Radioprotector or a Modulator of DNA-Repair Activity?

**DOI:** 10.3390/nu5020525

**Published:** 2013-02-07

**Authors:** Anupam Chatterjee

**Affiliations:** Moleculcar Genetics Laboratory, Department of Biotechnology & Bioinformatics, North-Eastern Hill University, Shillong 793022, India; E-Mail: chatterjeeanupam@hotmail.com or anupamchatterjee@nehu.ac.in; Tel.: +91-364-272-2403; Fax: +91-364-255-0076

**Keywords:** glutathione, radioprotector, glutathionylation, chromosome aberrations, DNA repair

## Abstract

The tripeptide glutathione (GSH) is the most abundant intracellular nonprotein thiol, and it is involved in many cellular functions including redox-homeostatic buffering. Cellular radiosensitivity has been shown to be inversely correlated to the endogenous level of GSH. On the other hand, controversy is raised with respect to its role in the field of radioprotection since GSH failed to provide consistent protection in several cases. Reports have been published that DNA repair in cells has a dependence on GSH. Subsequently, *S*-glutathionylation (forming mixed disulfides with the protein–sulfhydryl groups), a potent mechanism for posttranslational regulation of a variety of regulatory and metabolic proteins when there is a change in the celluar redox status (lower GSH/GSSG ratio), has received increased attention over the last decade. GSH, as a single agent, is found to affect DNA damage and repair, redox regulation and multiple cell signaling pathways. Thus, seemingly, GSH does not only act as a radioprotector against DNA damage induced by X-rays through glutathionylation, it may also act as a modulator of the DNA-repair activity. Judging by the number of publications within the last six years, it is obvious that the field of protein glutathionylation impinges on many aspects of biology, from regulation of protein function to roles of cell cycle and apoptosis. Aberrant protein glutathionylation and its association with cancer and other diseases is an area of increasing interest.

## Abbreviations

GRX: glutaredoxin
MPG: mercaptopropionylglycine
BSO: buthionine sulfoximine
NHEJ: non-homologous end-joining
AP-1: activator protein 1
NF-κB: nuclear factor kappa B
Sp-1: Specificity Protein 1
APE1: apurinic endonuclease 1
NPSH: non-protein bound sulphydryls
MEA: β-mercaptoethylamine
AET: *S*-2-aminoethylisothiourea
TRX: thioredoxin
SSb: single-strand break
dsb: double-strand break

## 1. Introduction

The tripeptide glutathione (L-γ-glutamyl-L-cysteinyl-glycine; GSH) is the most abundant intracellular nonprotein thiol that reaches millimolar concentrations in most cell types. It is present predominantly in a reduced form (GSH), which is biologically an active form. It is involved in many cellular functions, including antioxidant defense via direct interaction with reactive oxygen/nitrogen species (ROS/RNS) or via activities of detoxication enzymes like GSH peroxidases and GSH-*S*-transferases [1]. It has been suggested that antioxidants present in foods may work well as antioxidants *in vivo* and also bring beneficial health effects through other mechanisms, including acting as inducers of mechanisms related to antioxidant defense [2], longevity [3], cell maintenance, and DNA repair [4]. GSH is found in high concentrations in foods but it is degraded in the intestine and poorly absorbed by humans [5]. Over the years, a great deal of information has been gathered on the role of GSH in maintaining the intracellular reduction-oxidation (redox) environment that is critical for various cellular activities. Under oxidizing conditions, two molecules of GSH are linked by a disulfide bond to form the oxidized glutathione (GSSG), thus resulting in a decreased GSH-to-GSSG ratio. 

GSH has multiple roles in the regulation of cellular homeostasis. Antioxidative functions of GSH are expressed by either direct interaction with ROS [6] or donation of electrons to other redox systems, such as glutathione peroxidase (GPX) and glutaredoxin (GRX) [7]. In addition to antioxidation and electron donation, GSH is also required for maintaining homeostasis in animals, such as detoxification, by forming conjugates with toxicants and suppressing apoptosis [8,9]. GSH exerts its multiple functions mainly by two means, *i.e.*, enzymatic and non-enzymatic reactions (Table 1). Intracellular GSH is compartmentalized within the nucleus, mitochondria and endoplasmic reticulum, all of which constitute separate redox pools that are distinct from the cytoplasmic pool in terms of distribution of the GSH and GSSG forms, their redox potential and their control of cellular activities. The independent GSH pool of the nucleus plays an important role in protection against the oxidant and ionizing radiation-induced DNA damages [10] and in preserving nuclear proteins in a reducing environment for gene transcription during the cell cycle progression [11].

Exposure of cells to ionizing radiation results in production of ROS, which brings about depletion of cellular antioxidant stores, most prominently GSH [12]. Therefore, as an antioxidant, GSH has been considered a radioprotector. Increasing interest is being focused on the role of oxidizable endogenous thiols, particularly the reduced glutathione (GSH), in determining the inherent sensitivity of cells to various mutagens including radiation. It has been demonstrated that an aggressive tumor can be eliminated by using a therapy based on the modulation of GSH levels in cancer cells [13]. Many studies show the important role exerted by GSH in DNA synthesis and cell proliferation [14], regulation of the nuclear matrix organization [15] and maintenance of cystine residues on zinc-finger DNA-binding motifs in a reduced and functional state [16]. 

**Table 1:** Multiple functions exerted by glutathione.

Type of Reaction	GSH Acts as	Name of the Enzyme	Acts on	Citation
Enzymatic	electron donor	Glutathione peroxidase	various peroxides	[17]
Non-enzymatic	adduct (as in detoxification)	Glutathione *S*-transferase	xenobiotic compounds	[18]
	building block of leukotriene (LT)	LTC_4_ synthase	conjugate LTA_4_ to form LTC_4_ and finally LTD_4_	[19]
	antioxidant		reactive oxygen species	[20]
	reducing agent		cytochrome C	[21]
	hydrogen-donor		ribonucleotide	[22]
	oxidative and nitrosative modifier		SH-group of GSH	[23]
	oxidative modifier of proteins		Cys-SH of protein and GSH	[24]
	reversible glutathionylation		GSH moiety of GRX that can be freed by another GSH	[25]

This review is meant to give an overview of the potential role of cellular GSH as a protector of cellular damages caused by radiation or xenobiotic chemicals; and an attempt is also made to portray the current understanding of GSH as a radiomodifier. The review is organized into four sections, with the first addressing the role of GSH as a radioprotector and its controversy; the second addresses the involvement of GSH in reversible protein glutathionylation which is a common feature of redox signal transduction and regulation of activities of redox sensitive thiol-proteins; the third section reviews the available knowledge on the role of GSH in DNA repair; and the last and the fourth section pertains to reviewing the biological implication of glutathionylation.

## 2. Radioprotectors: Reducing Agents/Free Radical Scavengers

A large amount of chemical changes in biomolecules, especially DNA, are made by free radicals that are generated by mutagenic agents, including ionizing radiation [26]. However, most, if not all, of these changes are amenable to repair, either by chemical reactions or, at a later stage, by biological processes [27]. It is also true that the development of the field of radiation protection has been closely tied to the use of ionizing radiation ever since it was recognized that the latter could induce damage to biomolecules. It was reported by Dale for the first time who noted the effects of colloidal sulpher and thiourea on the responses of enzymes to X-rays. Since then, sulfhydryl compounds are known to play an important role in chemical protection of biological tissues from damaging effects of ionizing radiation. Therefore, for an agent to be a radioprotector, it must be present at the time of radiation to compete with the radicals produced through radical-scavenging mechanisms. It is well known that the radioprotectors can exert general antioxidant activity besides their ability to scavenge radicals; however, not all antioxidants afford radioprotection [28]. This difference may be attributed to the relative reactivity of radiation-induced reactive species compared to those generated under conditions of general oxidative stress (*i.e.*, H_2_O_2_ exposure). The hydroxyl radical (•OH) is truly the mediator of much of the radiation-induced DNA damages that are initiated by abstraction of a deoxyribose hydrogen atom by the hydroxyl radical [29]. Interestingly, it has also been reported that there is no relationship between the level of protection and the overall rate for •OH removal [30]. It was noted that protection occurs only with those •OH scavengers that are able to react and form secondary reducing radicals (alpha-hydroxy radicals, RCOH) [31]. Therefore, the failure to scavenge this less reactive secondary species with conventional antioxidants is attributed to nonaccumulation of antioxidants in proximity to the secondary radicals; otherwise, these antioxidants may not have any kinetic reactivity to effectively scavenge the less reactive secondary species. Thus, thiols such as amifostine and GSH have sufficient reactivity to efficiently scavenge •OH, as well as secondary radicals. 

### 2.1. Reduced Glutathione as a Radioprotector

Traditionally, GSH in the cell nucleus has been considered a protector against oxidative stress and radiation-induced damages in DNA and DNA-binding proteins [32]. Various studies on the role of reduced GSH as a protector in different systems are summarized in Table 2. It has been shown that an exogenous addition of GSH could effectively reduce radiation-induced micronuclei [33] and chromosome aberrations in different systems [34], including the muntjac lymphocyte cultures [35]. Buthionine sulfoximine (BSO) specifically depletes the endogenous GSH level by inhibiting the enzyme γ-glutamyl cysteine-synthetase and increases the cellular radiosensitivity [36]. It has been demonstrated that BSO effectively depletes GSH and sensitizes normal and cancer cells to radiation [37,38] and other chemotherapeutic agents [39].

In contrast to limited studies on the protective effect of GSH employing parameters of chromosome aberrations, data obtained on the role of GSH in the inherent cellular radiation protection mechanism are quite rich and informative. It has been demonstrated that GSH plays a significant role in the cellular detoxification process [40], regulates various enzymatic pathways by acting as a cofactor [41], and is involved in cell growth and replication processes [42]. The level of cellular radiosensitivity has been shown to be inversely correlated with the endogenous level of non-protein-bound sulfhydryls (NPSH), GSH being the major component of NPSH [43]. However, it has been proposed that GSH is critical for determining the cellular radiosensitivity when it is present within the cell nucleus, particularly close to DNA [44]. It has also been demonstrated that the treatment of cells with thiols like β-mercaptoethylamine (MEA), *S*-2-aminoethylisothiourea (AET) and cysteine cause an increase in the free GSH content in the cell by triggering a release of the nucleo-protein bound GSH [45]. In view of this, it has been proposed that the action of various thiol protectors can be attributed partly to their ability to elevate the cellular GSH level [40,46]. There are several reports suggesting that an increased radiosensitivity is associated with a defective GSH metabolism. Various diseases like 5-oxoprolinuria, leukemia and anemia have been reported to be associated with a defective GSH metabolism [47]. With the availability of GSH deficient (GSH^−^) human cells, which have only 6% GSH level of the proficient cells, it has been demonstrated that they are more sensitive to radiation than normal cells [48]. Depletion of the endogenous GSH level in GSH proficient normal cells has been further demonstrated to be more radiosensitive [49,50].

**Table 2:** Summary of the role of reduced GSH as a protector in different systems.

Type of treatment and Parameters	Radiation and Dose	Dose of GSH Mode of Treatment	Results	Citation
Whole body radiation, weights and histologic appearance of tissues	X-rays; 8 Gy	4 mg/g in mice subcutaneously	No protection of cellular damage, rapid regeneration of tissues	[51]
Chromosome aberrations in *Tradescantia* root tips	Co^60^-source; 50 Gy	0.001–0.3 mM	Significant reduction	[52]
Frequency of sex-linked lethal and translocation in *Drosophila melanogaster*	X-rays; 20 Gy	3.33 g/kg, injected	No protection	[53]
Marrow prophylaxis (C57BL × C3H) F1 mice	X-rays	1.6 g/kg	Increment of LD_50/30_ from 7.25 to 9.5 Gy	[54]
Frequency of sex-linked lethal and translocation in *Drosophila melanogaster*	X-rays; 20 Gy	1.65 mg/kg	Significant reduction of sex-linked lethal, slight reduction of translocation	[55]
Mitotic index and mitotic delay time in mammalian L-5 cells	X-rays; 2 Gy	20 mM	Recovery rate of mitotic index facilitated but no effect on mitotic delay time	[56]
Reversion of his-dependent *S. tiphimurium* Ames test	-	5–20 mM with and without liver/kidney S9-fraction	Increased number of revertants; positive for mutagenicity	[57]
Muntjac lymphocytes	X-rays; 2–4 Gy	10–25 mM	Consistent protection of deletions; inconsistent protection of exchanges at 3 and 4 Gy	[35]
Short-term radiation lethality, adult male mice	X-rays; 4 Gy	15 mg/kg	Cysteine, GSH & MPG less efficient radioprotectors than WR-2721	[58]
Polychromatic erythrocytes in mouse bone marrow, peripheral blood; micronuclei	X-rays; 6 Gy	400 mg/kg	Reduction in frequency of micronuclei induction	[33]

### 2.2. Controversy on the Role of GSH as a Radioprotector

It is interesting to note that in spite of the wider acceptance of the role of GSH as a radioprotector, there is some controversy with respect to its role in the field of radioprotection. As shown in Table 2, the reduced GSH did not provide any protection consistently in all cases. It was noted that radiation-induced translocations in *Drosophila* or chromosomal exchange aberrations in mammalian systems were not protected by GSH [35,40]. In another study, it has been shown that induction of GSH 100%–200% higher than its normal level provides only a small protection [59]. Moreover, radioprotection by GSH through hydrogen donation to DNA radicals was not found to be effective in oxygenated cells because the normal intracellular GSH concentration is not sufficient for effective competition with oxygen. Under hypoxic conditions, GSH becomes more competitive, and its depletion can markedly affect radiosensitivity [60].

Available evidence suggests that GSH may not be an efficient protector of DNA due to its −1 net charge, which, on the basis of counter-ion condensation and co-ion depletion phenomena, may allow its dissociation from DNA [61]. It was observed that chromosome aberrations induced by a radiation dose of 3Gy or above are not protected well by GSH pretreatment in mammalian lymphocytes [35]. A differential protection was demonstrated in a study, in which an elevated level of GSH decreased the frequency of radiation-induced deletions but increased the frequency of aberrations of the chromosome exchange type [38]. Radicals in the deoxyribose moiety of DNA formed under aerobic conditions are converted to peroxyl radicals through trapping by oxygen at a diffusion-controlled rate. From the point of view of radiation chemistry, it is demonstrated that GSH, as a major thiol compound in the cell, plays an important role in the conversion of DNA-derived peroxyl radicals to strand breaks [62,63]. Therefore, these results do not support GSH as radioprotector. 

In a separate study [64], an attempt was made, by employing the comet assay, to address whether or not the pretreatment with exogenous GSH protects or potentiates the yield of chromosomal damage induced by ionizing radiation. A roughly 20% increase in the endogenous GSH level was observed after a 3-h treatment with GSH exogenously, which could reduce the frequency of all types of chromosomal aberrations and aberrant metaphases induced by 1 and 2 Gy of X-rays and also decreased the tail in the comet assay, an indicative of radiation protection. Such uniform protection by GSH pretreatment was not visible when cells were exposed to higher doses of radiation. Interestingly, in GSH-depleted lymphocytes, the frequency of radiation-induced chromosomal aberrations was found to be increased in a non-uniform manner. 

Controversial reports are also there with regard to the role of GSH in the induction of apoptosis which is dependent on cell types and pro-apoptotic stimuli [65,66]. Such conflicting results in the literature preclude the conclusion of the role of GSH in either radiosensitization or radioprotection. In principle, modification of DNA repair could have a greater impact on radiation therapy than on the number of lesions produced by radiation [52]. On the basis of all the aforementioned reports, one can say that GSH does not act as an efficient radioprotector against DNA damage induced by higher doses of X-rays. However, there is an indication that GSH can also act as a modulator of DNA-repair activity.

## 3. *S*-Glutathioylation of Proteins: A Crucial Modulating Factor

**Figure 1:**
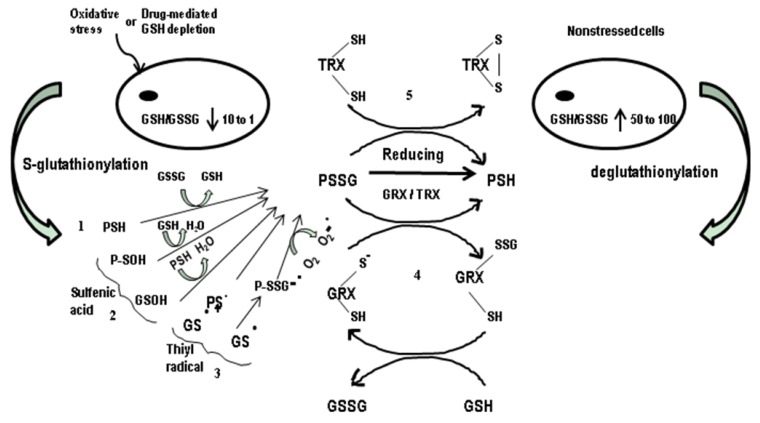
Schematic overview of protein–*S*-Glutathionylation and deglutathionylation. The figure depicts the different biochemical mechanisms by which protein thiol moieties could be converted to protein–SSG (PSSG) mixed disulfide adducts: (1) via thiol–disulfide exchange; (2) via sulfenic acid intermediates; (3) via thiyl radical intermediates. It also shows glutaredoxin-catalyzed and thioredoxin-catalyzed deglutathionylation of PSSG-mixed disulfides: (4) GRX (glutaredoxin) catalysis proceeding via a monothiol mechanism involving a selective double displacement reaction; (5) Thioredoxin (TRX) reduces oxidized proteins containing a disulfide bond and finally converted itself with disulfide bond.

Besides acting as a reactive oxygen scavenger, GSH can also form mixed disulfides with proteins, a process known as *S*-glutathionylation or reversible protein glutathionylation (Figure 1). The reversible formation of mixed disulfides between the oxidized glutathione and low-pKa cysteinyl residues is not only a cellular response to a mild oxidative/nitrosative stress, but also occurs under normal physiological conditions [67]. Cysteine is present in the active site of many proteins and in protein motifs that function in protein regulation, cellular signaling, and control of gene expression [68]. Oxidative modifications of the cysteine sulfhydryl group have received increased attention for the last decade. Such glutathionylation has been implicated as a mechanism in protection against the irreversible protein oxidation during oxidative stress [69]. A specific posttranslational modification of the protein cysteine residues occurs not only during the oxidative stress, but also in unstressed cells by the addition of GS^−^. Such oxidative thiol modifications lead to a change in the structure of proteins and cause either their activation (e.g., in hRas-Cys-118 [70] and mitochondrial glutathione S-transferase [71]) or inactivation (e.g., in phospho-fructokinase [72] and nuclear factor kappa B (NFκB) [73]).

In fact, *S*-glutathionylation is a prevalent protein modification; however, its mechanisms of protein–SSG (PSSG) formation are not yet resolved. Figure 1 depicts the different mechanisms of PSSG formation that may occur spontaneously or be catalyzed by enzymes that are yet to be identified. A large number of proteins have also been identified as potentially regulated by reversible *S*-glutathionylation. Deglutathionylation is the process by which GSH moiety can be removed from protein–mixed disulfides, and it occurs when the environment becomes more reducing in an enzyme-dependent or -independent manner (Figure 1). Enzymes capable of reducing *S*-glutathionylated proteins include glutaredoxins (GRX; also known as thioltransferases) and thioredoxins (TRX) [74]. Glutaredoxins (GRXs) are small redox enzymes of ~100 amino acid residues, which use GSH as a cofactor. Structurally, GRXs are very similar to thioredoxins, retaining the same fold and active sites. Both the TRX and GRX families contain a conserved -Cys-X-X-Cys- active site, which is important for their redox regulatory functions [74]. They catalyze the reduction of disulfide bonds and become concomitantly oxidized by forming an intramolecular disulfide in the -Cys-X-X-Cys- active site. The oxidized enzyme is then reduced by the respective TRX reductase/GSH [75]. Since glutathionylation affects the function of many proteins, the reverse process—deglutathionylation of specific proteins—has been implicated in regulation of cellular homeostasis in health and diseases [24].

## 4. Probable Involvement of Cellular Glutathione in DNA-Repair

There is a series of DNA-repair pathways that are implicated in correcting different types of DNA damage, including single-strand breaks (ssbs) and double-strand breaks (dsbs), base damages, and DNA adducts. A distinct DNA damage is repaired by different pathways, and there is also an overlap and interaction among the various pathways [76]. However, the number of proteins and factors involved in these pathways and their regulatory networks keep growing [77]. DNA-repair pathways play an important role in protecting the genome from damages caused by endogenous and exogenous DNA-damaging agents [78]. It was shown that DNA repair in cells has a dependence on GSH, because there is evidence of accumulation of DNA damage in the organs of mice that had a defect in GSH metabolism, thereby inducing low levels of GSH [79].

It was suggested that ionizing radiation-induced DNA dsbs are critical lesions, which, if unrepaired or misrepaired, can cause chromosome aberrations, cell death, as well as mutations and cell transformation [80,81]. In an attempt to clarify the possible role of GSH in biochemical repair processes, the extent of rejoining of radiation-induced ssbs was determined up to 1 h after the exposure [82], and it was observed that the repair systems involved in the rejoining of oxically and hypoxically induced ssbs differed from each other and that the former was clearly dependent upon GSH. Radiation-induced chromosome exchange aberrations are thought to arise as a consequence of illegitimate reunion (misrejoining) of free ends from different DNA dsbs [83]. Such misrejoining may be expected to depend on the number and proximity of the breaks. Therefore, the failure of either restitution or illegitimate reunion soon after the dsbs induction by radiation in BSO-treated GSH-depleted cells could lead to an increase in the frequency of deletion and chromatid aberrations [38]. This observation is important since the role of GSH has been clearly demonstrated in DNA synthesis under certain conditions and as a cofactor in enzymatic repair processes in the cell [42,84]. The involvement of GSH in DNA repair is further supported by two observations: (1) an increase in the frequency of chromosome exchange aberrations and a decrease in the frequency of deletions in GSH/GSH-ester post-treated human lymphocytes irradiated at 4 °C [38]; and (2) inhibition of unscheduled DNA synthesis in BSO-treated ovarian carcinoma cell line and replenishment of GSH in BSO-treated cells with GSH-monoethyl ester, which resulted in a complete recovery of DNA-repair activity [85].

It was demonstrated by Preston [86] that if the DNA damage produced by two agents is repaired at very different rates, then the probability of producing a synergistic effect on the aberration frequency becomes low. On the other hand, if the damage from both agents is repaired rapidly, then there is a high probability of producing a synergistic or interactive effect. Considering these possibilities, a study was performed with an aim to investigate the role of GSH in DNA dsbs-rejoining by treating the cells with γ-rays and bleomycin, since both these agents show similar and apparently rapid rates of damage repair [87]. The BSO-treated samples showed higher sensitivity to radiation than BSO-untreated ones. A combined treatment with Blem and radiation induced higher frequency of chromosome aberrations, in particular the exchange aberrations and interstitial deletions. However, such increased frequency of exchange aberrations was reduced drastically and the frequency of terminal deletions increased significantly when the combined treatment was given to BSO-pretreated cells. The consistent level of Ku70 protein in all the treated samples, with the level of Rad51 being undetected in G_o_-lymphocytes indicates the involvement of the non-homologous end joining (NHEJ) pathway in the misrejoining of DNA breaks. Usually, in quiescent mammalian cells, this process of misrejoining is prevalently taken up by the NHEJ process. It may be hypothesized that reduction in the frequency of exchange aberrations after exposure to Bleomycin + radiation combined treatment in BSO-treated samples indicates the involvement of the endogenous GSH in the illegitimate reunion of DNA dsbs [88].

**Figure 2:**
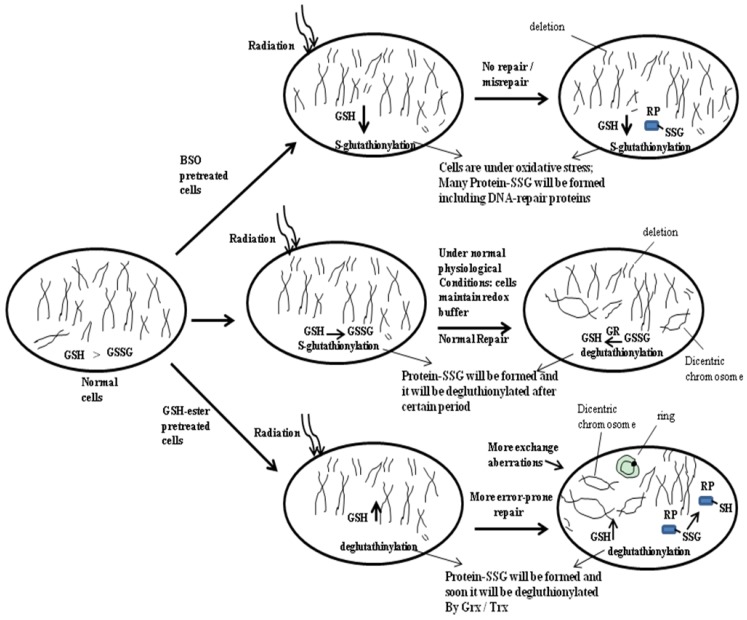
Radiation-induced DNA damage and repair that are regulated by cellular GSH level. This figure displays normal repair, no repair and more error-prone repair after irradiation to normal, BSO-pretreated and GSH-ester pretreated cells. A predictable role of glutathionylation and deglutathionylation of DNA-repair proteins (RP) in these three types of cells after radiation are depicted here schematically. RP-SSG, repair protein–disulfide; RP-SH, repair protein in reduced state; Grx, glutaredoxin; Trx, thioredoxin; GR, glutathione reductase.

It has been demonstrated that GSH may be an important determinant of a cell’s ability to repair DNA damage and resist cell death [89]. Ku is a multifunctional DNA-repair protein that exists as a heterodimer, consisting of Ku 70 and Ku 80 and plays an important role in the NHEJ repair pathway of DNA dsbs. There are reports that the Ku 70-SSG formation is responsible for the loss of Ku’s binding ability to DNA [90]. In another study, it was shown that, under oxidative stress conditions, Ku 70 and Ku 80 were degraded by the activated caspase-3 in the pancreatic acinar cells, whereas *S*-glutathionylation of procaspase-3 blocked its activation and regulated the activities of Ku indirectly [91]. It was demonstrated that the inhibition of Ku binding to DNA and leading to enhanced apoptosis was observed in G6PD-deficient cells, in which protein glutathionylation occurs more in response to the treatment with hydroxyethyl disulfide [90]. On the basis of the aforementioned information, one may hypothesize that glutathionylation of DNA-repair proteins may fail to repair or misrepair the DNA lesions induced by radiation in BSO-treated cells. On the other hand, a higher GSH-level leads to the deglutathionylation of DNA-repair proteins; thus, more repair or misrepair activities on DNA lesions are speculated (Figure 2).

Recently, it was reported that human apurinic/apyrimidinic (AP) endonuclease 1 (APE1) reduced its endonuclease activity due to *S*-glutathionylation of Cys99; the human APE1 is *S*-glutathinoylated under conditions of oxidative stress both in the presence of glutathione *in vitro* and *in vivo* [92].

## 5. Glutathionylation and Its Biological Implications, Particularly in Cancer

The ratio of the GSH/GSSG redox couple provides means of *S*-glutathionylation in a cell. Any modification of critical cysteine residues on enzymes, receptors, transport proteins, and transcription factors is recognized as an important mechanism of signal transduction and perturbation of the thiol–disulfide homeostasis is considered to be an important consequence of many diseases [93,94]. There are five criteria for evaluating redox regulation via reversible glutathionylation, *S*-glutathionylation must (a) be at a discrete site and functionally effective, (b) occur in a high GSH/GSSG ratio, *i.e.*, normal physiological conditions, (c) occur in response to a physiological stimulus, (d) occur through an efficient mechanism for specific protein–SSG formation, and (e) be reversed through an efficient mechanism (*i.e.*, deglutathionylation). 

Reports regarding protein glutathionylation are steadily increasing; however, they are yet to be documented in a physiologically relevant context. In many cases, protein glutathionylation was observed in an endogenous milieu, but its functional change and physiological impact were not studied. For example, in diabetic patients, glutathionylation of hemoglobin (HbSSG) has been reported. While an increase in HbSSG content is correlated with the increase in microangiopathy, whether or not this has any impact on redox homeostasis, or if it serves only as a biomarker of oxidative stress, is as yet unclear [93]. Glutathionylation of actin inhibits the actin polymerization, which contributes to the pathology of cardiac and skeletal muscles due to ischemia/oxidative stress and to the muscle pathology in Friedreich ataxia [94]. 

NF-κB and AP-1 are the two pleiotropic transcription factors that play an important role in almost all aspects of human cancers. NF-κB belongs to the Rel family and is comprised of diverse subunits to form hetero- or homo-dimers frequently including one subunit of p65 (relA) and another subunit such as p50, c-rel or relB [95]. The inactive form of NF-κB is localized in the cytoplasm through interaction with IκB repressor proteins [96]. Due to oxidative stimulation, IκB proteins are rapidly phosphorylated and degraded and thus set NF-κB free to enter the nucleus where it controls the transcription of a wide variety of genes [97]. NF-κB is negatively regulated via *S*-glutathionylation, which is overcome by glutaredoxin-1 (GRX1), the latter under physiological conditions catalyzes deglutathionylation and enhances NF-κB activation [73]. In another study, it was demonstrated that p50-NF-κB is susceptible to *S*-glutathionylation on Cys62, thereby inhibiting DNA binding [98]. Similarly, AP-1 (consisting of the members of *Jun* and *Fos* families) regulates the expression of a large number of genes. Klatt *et al.* reported that binding of AP-1-c-Jun subunit to DNA depended on the cellular GSH/GSSG ratio [99]. A decrease in GSH/GSSG ratio in a cell can induce *S*-glutathiolation of c-Jun at Cys269 and sterically blocks DNA binding.

The tumor suppressor gene p53 is mutated in many human cancers and has been known to play an important role in maintaining the genomic integrity, cell cycle control, DNA repair, differentiation and apoptosis. There are 10 cysteine residues in p53 protein, all of which are present within the DNA-binding domain. It was reported that in human colon cancer cells glutathionylation was identified at the proximal DNA binding domain (C124, C141 and C182) of p53 protein [100]. Glutathionylation of p53 is also a causative factor in inhibition of oligomerization and in subsequently preventing its transcriptional activation [100]. 

Apoptosis or the programmed cell death is a highly orchestrated and physiologically conserved mechanism of cell death. In the execution phase of apoptosis, a highly conserved family of proteins, the caspases (cysteine proteases), play an important role. The reversible *S*-glutathionylation of caspases has gained much interest as a sensitive mechanism for caspase activation in apoptotic signaling. Glutathionylation of caspase-3 inhibits its function in HL60 cells [101], whereas that deglutathionylation of caspase-3 activates the enzyme has also been demonstrated [102]. Reports are also suggesting that GSH specifically targets the proteolytic cleavage and activation of pro-caspase-8 [103]. It has been demonstrated that GRX is overexpressed in cancer cells [104] and protects the cell from apoptosis [105], while silencing the expression of GRX2 by RNAi sensitizes cells to apoptosis-inducing agents [106]. 

## 6. Conclusions and Perspectives

The most exciting development over the last decade has been the emergence of glutathione as an important signaling molecule. There are evidences that highlighted the important role of a variety of redox signaling mechanisms in the control of a plethora of cellular processes. Indeed, the list of cellular processes that are dependent on or influenced by GSH in biological systems continues to grow. *S*-glutathionylation, a redox-dependent posttranslational modification of a protein, accounts for the reversible regulation of the structure and function of a variety of proteins that are integral to cell structure, signaling, and metabolism. Therefore, the deleterious consequences that GSH depletion shows under oxidative stress are not only attributable to the lack of an antioxidant, but also to the blockade of *S*-glutathionylation-dependent signaling mechanisms, just as the ATP depletion can affect phosphorylation-mediated signaling.

The most obvious conclusion to be drawn from this review is that numerous agents can modify the radiation damage in more than one way. It is likely that all radiation response modifiers affect multiple aspects of biology that are of possible relevance to the outcome. It is striking to know that GSH as a single agent is found to affect DNA damage and repair, redox regulation, and multiple cell signaling pathways. There is a need to design new classes of radioprotectors for clinical use. For this, the critical prerequisite is to understand the metabolic differences between the normal tissue and tumor cells. New radioprotectors should act on mechanistic aspects of radiation action, particularly forming DNA strand breaks rather than focusing on distal steps in a cellular response, like transport of signaling molecule or induction of pathways leading to cell death, including autophagy, apoptosis, and necrosis. Clearly, many questions remain unanswered. Could deglutathionylation of nuclear proteins play a role in the interaction of DNA double-strand breaks leading to form higher frequencies of chromosomal exchanges in the presence of a higher level of GSH? If it is so, then cancer cells with higher levels of GSH [107] can form higher chromosome exchanges after radiation treatment, and such exchange-loaded cells could lead to apoptotic cell death [108]. It is probable that, the near future, we will be able to answer this and other intriguing questions. 
